# Whole-Cell Fluorescent Biosensors for Bioavailability and Biodegradation of Polychlorinated Biphenyls

**DOI:** 10.3390/s100201377

**Published:** 2010-02-21

**Authors:** Xuemei Liu, Kieran J. Germaine, David Ryan, David N. Dowling

**Affiliations:** Department of Science and Health, Institute of Technology Carlow, Kilkenny Road, Carlow, Ireland; E-Mails: Liux@itcarlow.ie (X.M.L.); germaink@itcarlow.ie (K.J.G.); david.ryan@itcarlow.ie (D.R.)

**Keywords:** biosensor, *Pseudomonas* F113, PCB, biodegradation

## Abstract

Whole-cell microbial biosensors are one of the newest molecular tools used in environmental monitoring. Such biosensors are constructed through fusing a reporter gene such as *lux*, *gfp* or *lacZ*, to a responsive promoter. There have been many reports of the applications of biosensors, particularly their use in assaying pollutant toxicity and bioavailability. This paper reviews the basic concepts behind the construction of whole-cell microbial biosensors for pollutant monitoring, and describes the applications of two such biosensors for detecting the bioavailability and biodegradation of Polychlorinated Biphenyls (PCBs).

## 1. Introduction

Environmental risk assessment is an essential tool in the investigation of polluted sites. Monitoring practices for assessing these risks usually involve the determination of the total concentration of pollutants using sophisticated chemical analytical techniques such as Gas Chromatography-Mass Spectroscopy (GC-MS) or High Performance Liquid Chromatography (HPLC) assays. The use of the total concentration is likely to overestimate the risk as only a fraction of the total amount of the pollutant, the bioavailable fraction, will actually have an impact on living organisms; this inability to differentiate between the two represents a major disadvantage of traditional analytical methods. This discrepancy between the total and the bioavailable fractions is particularly significant in the case of contaminants with poor aqueous solubility (e.g., PCBs, Poly Aromatic Hydrocarbons [PAHs]) [1]. The ability to monitor the bioavailability of a pollutant is essential, as it not only gives more accurate information regarding the risk that the contaminated site poses to human health, but also determines the effectiveness of potential bioremediation processes. Nowadays, increasing attention has been given to bioavailability assays that better predict the real exposure risks [2]. One such alternative is the use of biosensors which are highly selective and sensitive to a particular pollutant.

Whole-cell microbial biosensors have become one of the newest dimensions of molecular tools in environmental monitoring [3–5]. Microorganisms, due to their low cost, lifespan, and range of suitable pH and temperatures, have been widely employed as the biosensing elements in the construction of biosensors [6].

In the past decade, their applications were mainly focused in three areas:

- Monitoring survival and competition ability of bacteria [7–11].
- Monitoring plant root colonization of pollutant degrading bacteria in complex environmental samples [10,12–14].
- Monitoring the level of specific environmental pollutants [13,15–20].

**Figure 1.**
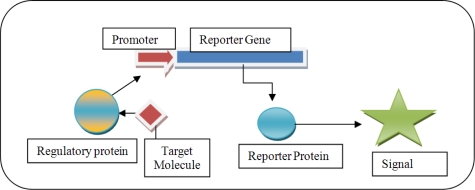
Graphic illustrating the concept of a whole-cell transcriptional biosensor.

In recent years, one of the most interesting areas utilising biosensor technology is the detection of environmental pollutant bioavailability, bioremediation, and toxicity. These biosensors are constructed by fusing a pollutant-responsive promoter to a reporter gene coding for a protein that can be easily quantified, and such constructs can be located on plasmids or on the chromosome (Figure 1). The efficacy of such biosensors was demonstrated by Willardson *et al.* [21]. The results they obtained showed that their toluene sensing, luciferase based whole-cell biosensor accurately reported toluene concentrations that were within the ±3% range as measured by standard GC-MS.

The biosensors rely on analysis of gene expression, typically by creating transcriptional fusions between a promoter of interest and the reporter gene. The extent of reporter gene expression may serve as a measure of the availability of specific pollutants in complex environments. Novel areas for applying these biosensors have been previously documented and include the construction of whole-cell biosensors as specific and sensitive devices for measuring biologically relevant concentrations of pollutants [4,15–18,21–27].

Previous applications of whole-cell microbial biosensors for environmental studies mainly concentrated on their use as biomarkers to investigate survival and competition ability [7–11] and as biosensors to detect the bioavailability or toxicity of environmental pollutants [15,16,28–33]. Layton *et al*. [34] reported a bioluminescent biosensor strain, *Ralstonia eutropha* ENV307 (pUTK60), detecting the bioavailability of PCBs by inserting the biphenyl promoter upstream of the bioluminescence genes. In the presence of biphenyl, bioluminescence was generated in a concentration-dependent manner. Kohler *et al*. [35] used an immobilized recombinant *E. coli* reporter to detect the bioavailability of 4-chlorobenzoate.

Compared with traditional detection methods for monitoring environmental pollutants, whole-cell biosensors provide the following advantages [36]:

- Biosensors determine only the bioavailable fraction of compounds, thus giving a more accurate response on the toxicity of a sample. Bioavailability is also important in bioremediation. If substances are bioavailable, they are potentially biodegradable.
- Biosensors provide an inexpensive and simple way of determining contaminants.
- As they are living organisms, they provide information on toxicology of different compounds.
- Some stress-induced biosensors report the mutagenic effects of samples with great sensitivity.
- Biosensors are unsurpassed in studying gene expression and physiology of bacteria in complex environments.

### 1.1. Commonly Used Reporter Genes

The reporter gene usually encodes an enzyme catalyzing a reaction that can be easily monitored. It determines the sensitivity and detection limits of the biosensor. Specific characteristics are needed for the reporter gene to be used in a biosensor. The gene must have an expression or activity that can be measured using a simple assay and it must reflect the level of chemical or physical change. Also, the biosensor must be free of any gene expression or activity similar to the desired gene expression or activity that is being measured to prevent misinterpretation of the response [37]. Several reporter genes meet the necessary requirements and are frequently used including *gfp*, *lacZ*, *lucFF*, *luxAB*, and *luxCDABE* with *gfp* and *luxCDABE* [29,38–40] being the most commonly used.

The *gfp* gene encoding Green Fluorescent Protein (GFP), originated from the jellyfish *Aequorea victoria* and its chromophore is assembled by the self-catalyzed covalent modification of amino acids Ser-Tyr-Gly at positions 65–67 to form a *p*-hydroxybenzylidene-imidazolidinone species [41,42]. The wild-type chromophore is excited with UV or blue light at 396 nm or 475 nm and emits green fluorescence at 508 nm [41]. The fluorescence of GFP can be monitored without the destruction of the biological sample [42,43]. A large collection of GFP derivatives have been constructed by the optimization of codon usage to alter the spectral properties of GFP for use in different organisms [41,44,45]. There are many examples of using different derivatives of GFPs in the construction of microbial biosensors for detecting environmental pollutants [46–50].

The bioluminescence gene *lux* cloned from *Vibrio fischeri*, *Photorhabdus luminescens* and others [51], coding for the enzyme luciferase, is another reporter gene regularly used for the construction of biosensors to monitor environmental pollutants. The light emitted by the labeled strain can be proportional to the concentration of the target pollutant. Bioluminescence has been used very successfully as a reporter for pollutant detection using sensitive instrumentation including fiber optic probes and integrated circuit chips detecting light production [52,53]. A comprehensive review of the application of bioluminescent genes and bacteria from 2000–2007 was reported by Girotti *et al*. [54].

Li *et al*. [26] constructed toluene bacterial biosensors which comprised of two reporters, *gfp* and *luxCDABE*. The bacterial luminescence biosensor allowed faster and more sensitive detection of toluene, while the fluorescence biosensor strain was much more stable and thus more applicable for long-term exposure.

### 1.2. Promoters and Regulatory Elements for the Construction of Biosensors

The selection of the promoter portion of the biosensor construct is dependent on the target molecule being monitored. A selected promoter sequence is normally placed at the 5′-region of the reporter system where it can be switched on in the presence of the target pollutant, thus turning on the expression of the reporter. The key factors when choosing promoters are sensitivity and specificity. Promoters often respond to groups of compounds rather than to a specific compound, and may also behave differently in different microorganisms. e.g., Winther-Larsen *et al*. [55] stated that the expression of the p_m_ promoter is substrate-dependent and host-specific (more details on this promoter are described in 2.1).

A variety of well-characterized promoters are available for the construction of pollutant-reporting biosensors. These promoters include those for hydrocarbons and organic solvents [56–60], various heavy metals [17,18,61–63], pesticides [64,65], salicylates [66], various organo-phosphorous nerve agents [67,68], and mutagens and genotoxins [69,70]. Promoters are also available for the evaluation of general toxicity [71–74].

One of the greatest limitations of whole-cell biosensor development is the availability of strong promoters that respond only to relevant stimuli. To circumvent this obstacle, more knowledge on gene regulatory networks in bacteria is needed. Linking metagenome information with the meta-transcriptome analysis of microbial communities using microarray technology could provide an immense source of new regulatory elements in the future [75]. Another option is to synthesize ‘super promoters’ based on consensus sequences obtained from comparative studies of different promoters in known regulatory networks [75].

## 2. Development of Biosensors to Detect PCB Biodegradation

PCBs were detected in the environment for the first time in 1966 by Jensen [76], and they have since been found all over the world including in Arctic and Antarctic regions [77]. The production of PCBs was banned in 1970 in the USA and in the Czech Republic in 1984 [78]. However, several hundred million kilograms has been released into the environment. Wiegel and Wu [79] documented that one-third of all US produced PCBs currently reside in the natural environment.

One of the major threats to public health from PCBs is that they accumulate within the food chain [80,81]. Contaminated fish consumption is a major route of PCB bioaccumulation in humans [82]. The bioaccumulation capability of PCBs in salmon has increased to a much higher extent than in other foods [83]. Traditional methods applied in the remediation of PCB contamination include incineration, vitrification, solidification/stabilization, solvent extraction, thermal desorption and land filling [84]. In the last decade, microbial-mediated degradation has been considered as one of the main processes in the alleviation of PCB pollution from contaminated environments [85].

Microorganisms which are capable of growing on biphenyl as sole carbon source were first isolated in 1970 [86,87]. In 1973, Ahmed and Focht [88] reported that *Achromobacter* degrades a few lightly chlorinated PCBs. Since then, numerous PCB-degrading bacterial strains have been isolated from PCB contaminated sites [89–95]. Nearly all of these isolates are able to degrade only two bi-chlorinated PCBs and very few bacteria have been found with the ability to degrade more highly chlorinated congeners [91,96]. These microorganisms belong to both Gram-negative and Gram-positive genera including *Pseudomonas*, *Burkholderia*, *Achromobacter*, *Comamonas*, *Ralstonia*, *Acinetobacter*, *Rhodococcus* and *Bacillus* [97–99].

**Figure 2.**
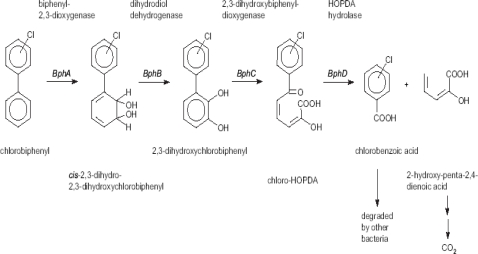
Biphenyl (*bph*) upper-pathway of aerobic PCB degradation [89,100]. Intermediates such as hydroxylated chlorobiphenyls [102] and chlorobenzoic acids [13,19,103] been used as targets in developing biosensors for monitoring PCB biodegradation.

PCBs are broken down by the catabolic “biphenyl upper pathway” or the “bph pathway” [96,100] which involves four enzymes: biphenyl 2,3-dioxygenase (BphA), cis-2,3-dihydro-2,3-dihydroxybiphenyl dehydrogenase (dihydrodiol dehydrogenase, BphB), 2,3-dihydroxybiphenyl 1,2-dioxygenase (BphC) and 2-hydroxy-6-phenylhexa-2,4-dienoate hydrolase (HOPDA Hydrolase, BphD). The biphenyl upper pathway breaks down biphenyl into benzoic acid and 2-hydroxy-penta-2, 4-dienoic acid [101] as shown in Figure 2. The aliphatic acid is metabolized via acetyl-CoA through the tricarboxylic acid cycle ultimately leading to CO_2_. The chlorobenzoic acids can be mineralized in co-culture with bacterial strains which can use chlorobenzoic acid as carbon source.

### 2.1. Biosensors Based on Monitoring Chlorobenzoic Acids

The p_m_ promoter is derived from the toluene degrading TOL plasmid and regulates the meta-cleavage pathway of aromatic hydrocarbon degradation [104]. The meta-pathway is induced by various benzoic acid derivatives and this induction is mediated by the substrate-activated XylS protein. The p_m_ promoter and its activator protein have previously been inserted into various vector systems and shown to be a useful expression system for controlled expression of recombinant proteins in several Gram-negative bacteria [105–111].

**Figure 3.**
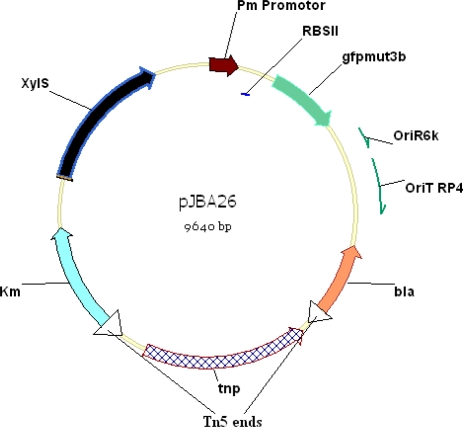
Genetic Map of pJBA26 vector [12], the region between the Tn5 ends from the kanamycin resistance gene (Km) to the β-lactamase gene (bla) can be transposed into the chromosome of suitable recipient cells such as *Pseudomonas fluorescens.*

The promoter-reporter fusion KmR-xylS-p_m_-gfpmut3* in plasmid pJBA26 (Figure 3) [12] has been used to construct a whole-cell biosensor to report PCB degradation. The *gfpmut3b* gene in plasmid pJBA26 is a variant of the wild-type *gfp* gene in which two amino acids have been substituted (S65G, S72A). These substitutions result in up to eight-fold enhanced fluorescent signal [112]. Gfp-mut3*, a mutant of *gfpmut3b*, was inserted in to a pUC18-NotI based cloning vector by introducing a SphI site in the start codon of *gfp-mut3b* during PCR amplification. The sequence was also changed during PCR so that the gfp-mut3* contained an arginine residue instead of serine at position 2. The construct gfp-mut3* maintained its intensively fluorescent signal, and with an estimated half life of one day *in vivo* [113].

Boldt *et al.* [13] constructed a number of *gfp*-based *P. fluorescens* F113PCB biosensors using fusions of the *Escherichia coli* rrnBP1 ribosomal promoter and the p_m_-xylS system introduced by pJBA26 with *gfp* genes. *P. fluorescens* F113 was originally isolated from the rhizosphere of sugar beet [114] and was found to be an excellent root colonizer. The genes responsible for PCB degradation from *Burkholderia xenovorans* LB400, termed the *bph* operon [115], were chromosomally inserted into a spontaneous rifampicin-resistant mutant of F113 creating *P. fluorescens* F113rifpcb [116]. The biosensors constructed by Boldt *et al.* [13] were shown to be able to monitor the single-cell localization and activity of *P. fluorescens* F113 colonizing alfalfa roots. The monitoring systems permitted non-destructive *in situ* detection of cells on the entire root system grown in both the presence and absence of 3-chlorobiphenyl [13].

**Figure 4.**
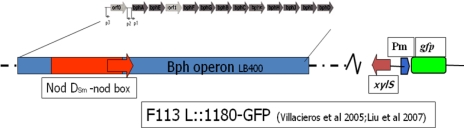
Genetic organization of whole cell rhizosphere biosensor *Pseudomonas fluorescens* F113L::1180-GFP. This bacterium can utilize biphenyl as a carbon source and biodegrade a range of PCBs. The *bph* operon originated from the PCB degrader *Burkholderia xenovorans* LB400 and it is constitutively expressed by the NodD-nod box regulatory unit from the symbiotic bacterium *Sinorhizobium meliloti*. The XylS-p_m_-gfp cassette originated from plasmid pJBA26. The host bacterium *P. fluorescens* F113 is an active plant root colonizer and biocontrol strain.

**Figure 5.**
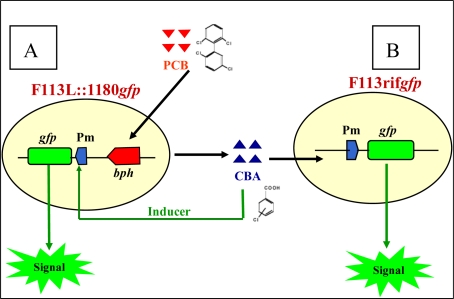
The use of two PCB biosensors. (A) F113L::1180*gfp* is able to report its own biodegradation of PCBs by switching on the signal, and the biodegradation of chlorobenzoic acid (CBA) by other CBA degraders by switching off the signal. (B) F113rif*gfp* is able to sense the biodegradation of PCBs by other PCB degraders by switching on the signal and the biodegradation of CBA by other CBA degraders by switching off the signal [13,19,103].

Liu *et al.* [19] constructed two further biosensors based on this system using a modified F113PCB strain (F113L::1180) (Figure 4) where the *bph* pathway was under the regulation of a strong constitutive promoter (Nod D1) (see Villacieros *et al.* [117] for details) with a corresponding higher level of *bph* gene expression and PCB transformation. A second biosensor (F113rif*gfp*), which cannot degrade PCBs, only responds to external chlorobenzoic acid derivatives, and by using these two biosensors PCB degradation could be detected *in vitro* and in soil (Figure 5). The sensitivity of this system towards 3-chlorobenzoic acid, 2,3-dichlorobenzoic acid and 3,5-dichlorobenzoic acid was found to be 0.1ppm using epi-fluorescent microscopy.

Expression of the p_m_ promoter is substrate-dependent and host-specific [55]. In a pure culture study [19], the p_m_ promoter was induced by 3-CBA in all of the biosensor strains and was greatly induced by 2,3-DiCBA, 3,5-DiCBA and to a lesser extent by 3,4-DiCBA as determined by spectrofluoremetry and epifluorescent microscopy. In addition, a linear relationship was observed between the fluorescent intensity and the concentration of 3-CBA, 2,3-DiCBA and 3,5-DiCBA ranging from 4 to 400 ppm. Previous studies used an immobilized recombinant *E. coli* reporter to detect the bioavailability of 4-chlorobenzoate [35]. This work shows that the biosensors have the potential to detect the bioavailability of other CBA derivatives, which are produced by the intrinsic biodegradation of PCBs in the environment.

## 3. Encapsulation of Biosensors for Environmental Use

Whole-cell biosensors provide effective tools for detecting environmental pollution and toxicity. Important aspects of biosensing environmental pollutants are the ability to monitor *in situ* and preferably on-line. One of the main problems is the difficulty of maintaining constant sensing activity and variability for extended periods at room temperature. To meet these demands, various conservation techniques have been reported including freeze drying, vacuum drying, continuous cultivation, and immobilization in biocompatible polymers of organic or inorganic origin [72,118,119].

Encapsulating whole-cell biosensors in natural or synthetic polymers has been shown to be useful for the detection of environmental pollutants [120–123]. Polymeric matrices can provide a hydrated environment containing the nutrients and cofactors needed for cellular activity and growth. In addition, encapsulated cells are protected from toxic substances in their environment and maintain increased plasmid stability [124].

The following encapsulation parameters show the potential usefulness for developing an *in situ* application of whole-cell biosensors [53]:

- Agar/agarose: competent cells can be added to molten agar or agarose (1–5%). Gelation occurs as the agar or agarose cools to room temperature [125].
- Carrageenan: a 2% solution of carrageenan is warmed to 70–80 °C to initiate dissolution and then maintained at 35–50 °C. The cell culture is also warmed and added to the carrageenan solution. Gel formation occurs through the addition of cold 0.1 M potassium chloride [124].
- Alginate: cells are added to a 1–8% solution of alginate; addition of 0.5 M calcium chloride or 0.1 M strontium chloride causes polymerization [124]. The bioluminescent bioreporter *P. fluorescens* HK44 has been immobilized on the end of liquid light guides using this method [126–127].
- Polyurethane–polycarbomyl sulfonate (PCS): polyurethane or PCS at a polymer content of 30–50% is mixed with a 1% calcium-chloride solution, the pH is adjusted to approximately 6.5 and the cell mass is added. This mixture is sprayed into 0.75% calcium alginate, resulting in bead formation. After one hour, the beads are removed, washed and introduced into a 2% sodium-tripolyphosphate buffer, which dissolves the alginate layer leaving only a layer of polyurethane–PCS surrounding the cells [126].
- Polyacrylamide: cells are mixed in a solution of acrylamide and bisacrylamide. Ammonium persulfate and N,N,N′,N′-tetramethylethylenediamine (TEMED) are then added to initiate polymerization [128].
- Polyvinyl alcohol: the cell suspension is mixed with a 13% polyvinyl alcohol, 0.02% sodium-alginate mixture. Gel formation occurs on contact with a solution of saturated boric acid and 2% calcium chloride [129].
- Sol–gel: cells are combined with 0.1 M Tris-Cl and tetramethylorthosilicate, tetraethoxysilane, methyltrimethoxysilane, ethyltrimethoxysilane, propyltrimeth
- Oxysilane or polydimethylsiloxane. Solidification times vary depending on the concentrations used [130].
- Polyethylleneimine [131].

Alginate had been previously applied as a delivery system for *Pseudomonas fluorescens* F113*lac*ZY in sugar-beet root colonization experiments [132]. It was shown that cells encapsulated in alginate polymers displayed more efficient root colonization and had a longer shelf life (up to eight weeks of storage), regardless of the conditions of incubation. Another F113 derivative F113rifPCB had also been alginate encapsulated and up to 100% of the encapsulated cells were found to be viable after 250 d storage [133].

### 3.1. Monitoring PCB Degradation in Vitro and in Soil Using Encapsulated PCB Biosensors

Liu [103] encapsulated two PCB/CBA biosensors (F113L::1180*gfp or* F113rif*gfp)* in alginate beads and examined the response of the biosensor cells to various PCBs and chlorinated benzoates in liquid cultures and in soil.

In liquid culture spiked with 3-chlorobiphenyl, 100% of the alginate encapsulated F113L::1180*gfp* biosensor cells were fluorescent after five days indicating that this strain was actively degrading the 3-chlorobiphenyl. In a similar soil based experiment, more than 30% of the alginate encapsulated F113L::1180*gfp* cells were visualized as *gfp*-expressing cells after 10 days. There was no other source of chlorobenzoates to induce this biosensor other than those derived from F113L::1180*gfp* own PCB degradation activity. This was demonstrated by the fact that when encapsulated F113rif*gfp* (a non PCB degrading chlorobenzoate biosensor) was inoculated under the same conditions no fluorescent cells were found.

**Figure 6.**
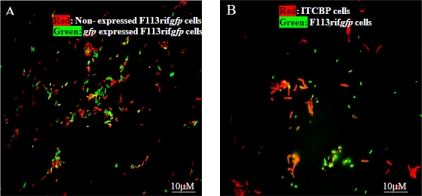
Epifluorescent micrographs showing encapsulated biosensor F113rif*gfp* detecting 3-CBP degradation by *Rhodococcus sp.* ITCBP. (A) Biosensor F113rif*gfp* cells in liquid culture; (B) *Rhodococcus* sp. ITCBP cells and biosensor F113rif*gfp* cells in the soil.

When the encapsulated F113rif*gfp* biosensor was co-introduced into 3-chlorobiphenyl liquid culture with the natural PCB degrader *Rhodococcus* sp. ITCBP [134] about 80% of the biosensor cells were visualized as *gfp*-expressing cells after five days (Figure 6A). In soil, 50% of the encapsulated biosensor cells were fluorescent after 10 days when co- inoculated with *Rhodococcus* sp. ITCBP (Figure 6B). No fluorescent cells were visualized in the control experiment using a pure culture of either F113rif*gfp* or *Rhodococcus* sp. ITCBP, thus validating the premise that the encapsulated biosensor, F113rif*gfp*, could be used to detect PCB degradation by other bacteria, in this case *Rhodococcus* sp. ITCBP.

### 3.2. Monitoring Chlorobenzoic Acids (CBA) Bioavailability and Biodegradation

When the encapsulated biosensors (F113L::1180*gfp* or F113rif*gfp)* were inoculated in minimal media broth or sterile soil supplemented with 0.1 mM 3-CBA or 2,3-CBA or 3,5-CBA, 100% of the cells were fluorescent after 24 hours. This result indicated that the encapsulation of the biosensors in alginate did not interfere with their CBA sensing ability either in liquid culture or in soil.

**Figure 7.**
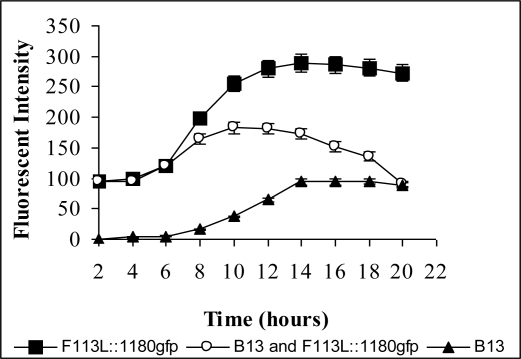
Biosensor detecting 3-CBA degradation by *Pseudomonas sp*. B13. Gfp fluorescent signal detected using a spectrofluorimeter. Note in the mixed cultivation, fluorescence decreases after 10 hours due to removal/degradation of 3-CBA by *Pseudomonas sp.* B13.

In 1 mM 3-CBA liquid culture, when either of the encapsulated biosensors (F113L::1180*gfp* or F113rif*gfp)* were co-inoculated with the chlorobenzoate degrader *Pseudomona*s sp. B13 [135] 80% of the biosensor cells were fluorescent after 10 hours. After 22 hours no fluorescent cells could be visualized (Figure 7). When the encapsulated biosensors were introduced into 3-CBA spiked soil previously inoculated with *Pseudomona*s sp. B13, the percentage of fluorescent cells dropped over time and after 10 days there were no fluorescent cells detected. These results demonstrated that the encapsulated biosensors could detect the biodegradation of chlorobenzoic acids by CBA degrading bacteria, in this case by *Pseudomonas* sp. B13.

## 4. Discussion and Conclusions

The majority of promoter-reporter biosensor systems currently being used are the result of cloning of a promoter upstream of a reporter gene cassette and the subsequent transfer of the plasmid construct into specific strains [30–32,34]. However, loss of these plasmids due to the starvation [136] and reduction in expression of the reporter gene due to multiple copies of the promoter binding region on the plasmid [137] poses problems when these biosensors are applied to *in vivo* situations. There are few reports of biosensors based on the chromosomal insertion of the promoter-reporter gene which produce more stable systems [16,128]. Liu [103] describes the construction of three chromosome-based biosensors *P. fluorescens* F113rif*gfp*, *P. fluorescens* F113rifPCB*gfp* and *P. fluorescens* F113L::1180*gfp*. The insertion of the *gfp* construct into the chromosome of these bacteria was confirmed by PCR and Southern blotting.

Studies have shown that the integration of the *gfp* reporter gene into the chromosome affected the growth ability of *Ralstonia eutropha* on 2, 4-dichlorophenoxyacetic acid [138] and on biphenyl [11]. It was important to ensure that the chromosomal insertion of the *gfp* construct did not affect any major catabolic pathway of these biosensor strains. Phenotypic characterization of F113rif*gfp*, F113rifPCB*gfp* and F113L::1180*gfp* by comparing the growth curve and Biolog^®^ Gram Negative (GN) profiles of the three biosensor strains to their parental strains showed there was no detectable disruption of major metabolic pathways by the insertion of this *gfp* construct. Rhizosphere colonization ability was not affected in the biosensors as green fluorescent cells colonizing pea (*Pisum sativum*) root was observed by Epifluorescent microscopy. Also the *bph* operon was not affected by insertion of the *gfp* construct, as documented by Southern blotting using a Digoxigenin (DIG)-labeling *bphC* probe [19]. While random chromosomal insertion offers many advantages over plasmid-based construction of sensors, the ideal approach for commercial applications would be the use of targeted insertion to known chromosomal sites.

Environmental contamination by chlorobenzoic acids has occurred as a result of decades of excessive use of herbicides (e.g., 2,3,6- trichlorobenzoic acid) and through partial degradation of other xenobiotic compounds, such as PCBs [139]. One of the clear impacts of chlorobenzoates on the environment is that they inhibit PCB metabolism [140,141]. It is essential to develop an easy and cost effective method to detect and remove these compounds from contaminated environments.

Whole-cell biosensor technology has been applied to detect the bioavailability or toxicity of environmental pollutants [4,36,142]. However, to our knowledge there are no studies to show the *in vivo* application of whole-cell biosensors to detect the biodegradation and decontamination of an environmental pollutant. Through the introduction of the biosensor F113L::1180*gfp* strain into PCB amended soil, PCB degradation could be monitored by observing *gfp* fluorescence in the cells. Another biosensor strain F113rif*gfp* could detect PCB degradation by other PCB degrader strains e.g., *Rhodococcus* sp. ITCBP. Potentially, this biosensor can be used to evaluate the PCB degrading ability *in situ* of intrinsic PCB degrading strains.

Previous studies had shown that the co-culture of PCB and CBA degrading strains led to the complete mineralization of PCB contamination from the environment [143–145]. The work detailed here showed that the PCB mineralization process can be monitored using the constructed biosensor strain F113L::1180*gfp*. When both the biosensor *P. fluorescens* F113L::1180*gfp* and *Pseudomonas* sp. B13 were co-inoculated into 3-CBP media, fluorescent cells were observed indicating that this biosensor detected its own biodegradation of PCBs. The percentage of fluorescent cells dropped to below detectable levels at the end of experiment, indicating that this biosensor also detected the mineralization of CBA by *Pseudomonas* sp. B13. Therefore, it is possible to monitor the complete mineralization process of PCB *in situ* using this biosensor.

It was also observed that the *gfp* gene was unevenly expressed within the biosensor populations. This is probably due to the spatial and temporal distribution of CBA compounds within the samples leading to the uneven expression of the p_m_ promoter within the populations [30,55].

Previous studies have shown that using immobilized biosensor cells to easily and accurately detect the availability of pollutants based on induction and *de novo* synthesis of reporter proteins is possible [15,52,146,147]. Boldt *et al.* [13] reported using F113rifpcb*gfp* to detect the biodegradation of 3-CBP and root colonization in 3-CBP spiked soil. However, only 1% of the bacterial cells were shown to be Gfp fluorescent. In this study, it was suggested that the presence of PCB or CBA degraders would affect the accuracy of the biosensor. Thus, an alginate bead immobilized system for the delivery and examination of the biosensors was developed. The evaluation of alginate as a carrier was studied previously and the results indicated a long survival of the genetically modified strain F113lacZY [132].

In addition to the longer survival and easy release advantages of encapsulating the cells in an alginate polymer, there is also some evidence in the study carried out by Liu [103] to show that higher levels of *gfp* expression could be achieved by encapsulating the biosensor strains which in turn lead to more accurate results. This study showed that:

In 3-CBP media co-inoculated with F113L::1180*gfp* and *Pseudomonas* B13, more fluorescent cells were observed using the encapsulated cells than when using the free cells. This indicated that the alginate provided a protective barrier to maintain the CBA breakdown products from PCB degradation by F113L::1180*gfp* within the beads. These increased levels of CBAs then induced the biosensor so that it could detect the occurrence of this PCB breakdown process accurately. When F113L::1180*gfp* was applied as free cells to PCB contaminated environmental samples, only 1% of the cells showed fluorescence compared to 10%–43% cells that showed fluorescence when the encapsulated method was used.

It should be remembered that in nearly all cases whole-cell biosensors are not applied *in situ* to soil and water but rather samples are taken from polluted sites and incubated in *in vitro* assays. This may not precisely reflect the real conditions in nature with respect to prevailing environmental conditions. The development of robust permeable sealed immobilised biosensor systems could be useful to overcome this challenge. The presence of auto-fluorescence within the sample matrix can also be a problem and attempts to address this issue have included the use of reporters such as urophorphyrinogen II methyl transferase (UMT), using the CobA gene as a reporter, which converts UMT to two fluorescent compounds. A whole-cell biosensor for arsenate detection was developed using this approach (148). An alternate approach is to counter-stain with methyl violet to limit the interference of auto-fluorescence as reported by Germaine *et al*. (14).

The presence of toxic compounds (both target and non-target) in the sample matrix may have detrimental effects on the host cell with respect to viability, physiological state and survival may have negative impact on the function of the sensor. In many cases these issues can be addressed by the appropriate choice and detailed evaluation of the host strain under different polluted soils.

Whole-cell microbial biosensors offer excellent possibilities for assaying the complex nature of bioavailable and bioaccessible fractions in thousands of cases of severe and toxic pollution, which currently cannot be easily addressed [2] and in general there is good correlation between such sensors and standard quantitative methods. In the case of the PCB biosensor described here, preliminary data indicated a strong positive correlation between the number of fluorescent cells and concentration of PCBs in soils or sediments as measured by standard analytical methods. The work detailed in this review suggests that encapsulated biosensors have real potential to detect the bioavailability and biodegradation of PCBs in the environment.
